# Assessment of Antioxidant Stability of Meat Pâté with *Allium cepa* Husk Extract

**DOI:** 10.3390/antiox12051103

**Published:** 2023-05-16

**Authors:** Irina Chernukha, Nadezhda Kupaeva, Daniil Khvostov, Yuliya Bogdanova, Jutta Smirnova, Elena Kotenkova

**Affiliations:** 1 V. M. Gorbatov Federal Research Center for Food Systems, Experimental Clinic and Research Laboratory for Bioactive Substances of Animal Origin, Talalikhina St., 26, 109316 Moscow, Russia; imcher@inbox.ru (I.C.); ju-deeschka@yandex.ru (J.S.); 2 V. M. Gorbatov Federal Research Centre for Food Systems of RAS, Laboratory of Molecular Biology and Bioinformatics, Talalikhina St., 26, 109316 Moscow, Russia; d.hvostov@fncps.ru; 3 V. M. Gorbatov Federal Research Centre for Food Systems of RAS, Department of Scientific, Applied and Technological Developments, Talalikhina St., 26, 109316 Moscow, Russia; yu.bogdanova@fncps.ru

**Keywords:** natural antioxidants, onion, total antioxidant capacity, pâté processing, quercetin, yellow onion husk

## Abstract

Antioxidants play a very important role in the food industry. Recently, both science and industry have shown substantial preference for natural antioxidants, including searching for antioxidant substances from natural sources without undesirable side effects. The purpose of this study was to evaluate the effect of adding *Allium cepa* husk extract at a volume of 68 or 34 µL/g of unsalted blanched materials to replace 34% and 17% of the beef broth, respectively, which corresponded to a total antioxidant capacity (TAC) of 44.4 or 22.2 µmol-equiv. Q/100 g meat pâté (i.e., 13.42 or 6.71 mg of quercetin/100 g meat pâté), on the quality and safety indicators of the developed meat pâté. The TAC according to a ferric reducing antioxidant power assay, thiobarbituric acid reactive substances, and physicochemical and microbiological characteristics were determined during the storage of the meat pâté. Proximal and UPLC-ESI-Q-TOF-MS analyses were also performed. The addition of yellow onion husk ethanolic extract to the meat pâté at both volumes allowed the maintenance of an increased content of antioxidants, which contributed to a decrease in the generation of secondary products of lipid peroxidation for 14 days of storage at 4 °C. The results of the microbiological analyses showed that the developed meat pâtés were safe according to all indicators of microbial spoilage within 10 days of production. The results supported the use of yellow onion husk extract in the food industry to contribute to improving the functionality of meat products, developing products for a healthy lifestyle, and providing clean-label foods without or with a minimal content of synthetic additives.

## 1. Introduction

Food products are important for maintaining many functions in humans, such as energy production; the supply of nutrients, including proteins and macro- and micronutrients; the maintenance of various metabolic processes; and the growth of the body [1]. A relationship exists between the food one consumes and one’s health [2], which promotes the development of functional food products that are characterized by scientifically proven biological activities and exert a beneficial physiological effect [3]. Food products can be made functional through fortification with natural (non-modified) ingredients and the addition, removal, or modification of the recipe by technological or biotechnological methods [4].

Meat and meat-containing products are one of the main food groups with high biological value, providing proteins and minerals—in particular, zinc and iron [5]. In addition, meat and meat-based products have a variety of preventive and health-promoting benefits [6], and meat is one of the most important food products along with vegetables, fruits, dairy products, and fish [7]. Therefore, meat is an acceptable matrix for functional food development [6]. However, the meat matrix is one of the most difficult for creating functional food products due to its complex composition and propensity toward oxidation and microbial spoilage [7]. The meat industry offers three options for the development of functional products: modifications at the farm level, raw materials, and meat products [8]. The beneficial properties of meat products can be improved by adding biologically active substances or probiotics, eliminating or replacing synthetic additives, and reducing cholesterol and energy levels [6,8]. Thus, a promising direction for the creation of functional meat products is the replacement of synthetic antioxidants with natural alternatives by enriching the product with plant antioxidants.

Liver and meat pâtés are cooked food products that have historically been considered traditional meals and are wide-spread in many countries, especially in Europe. The main ingredients of a pâté are liver, meat, fat, water, salt, various spices, and synthetic antioxidants [9,10]. Usually, pâtés are characterized by a high content of saturated fatty acids, fat, and non-heme iron and a low concentration of natural antioxidants. In addition, the intensive mincing of raw materials during pâté processing increases the sensitivity of lipids and proteins to oxidation [9,11]. Lipid oxidation is the main non-microbial cause of the deterioration of meat and meat products. Lipid oxidation is a very complex process involving many mechanisms interacting with each other. In brief, unsaturated fatty acids react with molecular oxygen by a free-radical mechanism [12]. During oxidative processes, hydroperoxides are generated, which are subject to further oxidation or decomposition with the formation of secondary reactive products such as aldehydes, ketones, acids, and alcohols. The presence of these compounds leads to a decrease in the quality and shelf life of meat products in terms of color, taste, aroma, texture, and nutritional value [10,13,14,15,16]. In addition, one of the most important problems of lipid oxidation is the generation of harmful compounds that could cause diseases, including atherosclerosis, cancer, inflammation, and aging processes stimulation [17,18,19]. The use of antioxidants in meat and meat-based products is the main way to slow down their oxidation in order to extend their shelf lives [10,13]. The antioxidants used in the food industry can be divided into natural and synthetic antioxidants. Butylated hydroxyanisole (BHA), butylated hydroxytoluene (BHT), and tertiary butylhydroquinone (THBQ) are the most widely used and studied [16,20], as well as ascorbic acid and tocopherol. Although both types of antioxidants play a very important role in the food industry, both science and industry have recently shown substantial preference for natural antioxidants, including searching for antioxidant substances from natural sources without undesirable side effects [21,22,23]. This trend is explained by the fact that synthetic antioxidants at high concentrations can have a negative impact on health [21,24], as well as the increase in consumer demand for “natural” products and products “without preservatives” [21].

Thus, plants, vegetables, fruits, and their co-products are a rich source of antioxidant compounds with good protective and therapeutic properties regarding socially significant diseases [13]. The use of such antioxidant compounds in the form of extracts is recommended, since purified phenolic compounds are more expensive. Moreover, extracts may have better antioxidant activity than pure compounds due to the potential synergistic effect [25]. *Allium cepa* L. (onion) was one of the first crops to be cultivated in the world, and it is currently one of the most popular [26], which is explained by its universal culinary use as a raw food or in various cooked forms: baked, boiled, stewed, grilled, fried, etc. [27]. In addition, onions are used in various forms of traditional medicine [28,29]. Onions have antimicrobial, antibacterial, anti-inflammatory, and antioxidant properties and present a bright taste and aroma; therefore, this plant is widely used in food and for the treatment of many diseases [30]. Numerous studies have confirmed that regular onion consumption reduces the risk of cancer, cardiovascular and neurodegenerative diseases, and osteoporosis [27,31]. The biological and medical benefits of onions are mainly associated with the high content of thiosulfinates and other organosulfur compounds. Besides well-known onions, several other species are also actively grown for culinary use, such as *Allium porrum* L., *Allium fistulosum* L., *Allium ascalonicum Hort.*, *Allium schoenoprasum* L., and *Allium tuberosum* L. [28,29].

We previously showed that the husks of red and yellow onions are a promising and rich source of natural antioxidants, especially quercetin and its glycosides [32]. By the method of kinetic chemiluminescence, we also found that the antioxidant compounds of yellow onion husk belong to three categories, according their power. Unlike red onion husk, which had the highest total antioxidant capacity, yellow onion husk contained almost equal amounts of strong, medium, and weak antioxidants, which suggested that this extract exerted a more uniform and long-lasting antioxidant effect. In addition, we determined that the long-term consumption of yellow onion husk extract improved the antioxidant status of aging rodents [33]. The availability and cheapness of this waste support the prospect of using onion husks as a source of natural antioxidants. Despite the substantial number of available scientific papers devoted to studying the qualitative composition of onion husks and bulbs and the effectiveness of various extraction methods [34], works addressing the preservation of the husk compounds’ antioxidant properties during meat product processing are difficult to find.

Thus, the purpose of this study was to assess the stability of the antioxidant properties of yellow onion husk ethanolic extract in a meat matrix, as well as to evaluate the effect of the added extract on the quality and safety indicators of the developed meat pâté containing beef and pork.

## 2. Materials and Methods

### 2.1. Chemicals

Quercetin (purity ≥ 95%, Bangalore, Karnataka, India) and iron (III) chloride hexahydrate (purity ≥ 99%, Taufkirchen, Germany) were purchased from Sigma-Aldrich (Saint Louis, MO, USA). Dipotassium hydrogen phosphate anhydrous (purity ≥ 98%), potassium dihydrogen phosphate (purity ≥ 98%), sodium acetate anhydrous (purity ≥ 99%), hydrochloric acid (HCl, 37%), thiobarbituric acid (purity ≥ 98%), 1-butanol (purity ≥ 99.5%), and formic acid (FA, purity ≥ 98%) were purchased from PanReac AppliChem (Darmstadt, Germany). Furthermore, 2,4,4-tris(2-pyridyl)-1,3,5-triazine (TPTZ, purity ≥ 96%) was purchased from BLDpharm (Shanghai, China). Acetic acid (purity ≥ 99.8%) and ortho-phosphoric acid (purity ≥ 85%) were purchased from Component-Reaktiv (Moscow, Russia). Sodium thiosulfate standard titer was purchased from LLC Himtitry (Pereslavl-Zalessky, Russia). Starch indicator and sodium sulphate anhydrous were purchased from Himmed (Moscow, Russia). Acetic acid was purchased from Labtech (Moscow, Russia), and potassium iodide was purchased from Reahim (Moscow, Russia). Deionized water for chromatography (18 W) was obtained using a Milli-Q Merck water purification system (Millipore, Darmstadt, Germany).

### 2.2. Preparation of Onion Husk Extract

For the preparation of yellow onion (*Allium cepa*) husk ethanolic extract (OHE), onions were obtained from the supermarket, producer OOO “Agroleto”, Krasnodar, Russia. The husks were ground (particle size 5 mm or less) and soaked in 70% ethanol (60 g/900 mL) for 24 h with gentle shaking at room temperature (22 ± 2 °C). The mixture was filtered through a paper filter and kept in an airtight bottle in a refrigerator at 4 °C until use. The total antioxidant capacity (TAC) of the OHE was 6.53 ± 0.18 mmol-equiv. Q/L.

### 2.3. Manufacture of Meat Pâtés

Meat pâtés were produced in the Department of Scientific, Applied and Technological Developments of V. M. Gorbatov Federal Research Centre for Food Systems of RAS according to the recipes indicated in Table 1. OHE was added instead of broth at volumes of 68 µL/g for experiment 1 (E1) and 34 µL/g for experiment 1 (E2), which corresponded to a TAC values of 44.4 and 22.2 µmol-equiv. Q/100 g meat pâté, respectively (or 13.42 and 6.71 mg of quercetin/100 g meat pâté).

Raw materials were cut into 200–300 g pieces and blanched separately in water at a temperature of 95 ± 5 °C: beef liver and lean pork for 15–20 min, pork heart for 120 min, and beef flank for 40 min. Onions were peeled and ground in a Bosch MCM3501M food processor (Bosch, Škofja Loka, Slovenia) with a power of 800 W and blanched with the addition of oil in a frying pan until fully cooked. Then, the heat-treated raw meat materials and onions were individually ground in a meat grinder Hurakan HKN-12SC (Hurakan, Guangzhou, China) to a particle size of 2–3 mm and homogenized in a cutter (Robot-Coupe, Montceau-les-Mines, France) at 3000 rpm for 5 min. The ingredients were added during final homogenization in the following order: minced beef flank, lean pork, and pork heart; minced beef liver; fried onion and dry ingredients; and beef broth. This product constituted the control sample for the experimental variants. The technological variance factor, which changed the composition and quality of the final products, was the replacement of 34% (E1) and 17% (E2) of the beef broth with OHE. The final temperature of the product at the end of homogenization was over 40 °C. The product was packed at 100 ± 1 g in vacuum packaging VakumPak-M (Webomatic, Bochum, Germany), PA/PE, size 150 × 200 mm, thickness 70 µm; cooked at a temperature of 72 °C in the geometric center of the bar; and then cooled to 4 °C in a water bath (EKROS 4310, Saint Petersburg, Russia) for 20–30 min and stored at 4 °C. The temperature control was carried out using a digital thermometer WT-1 (Xuzhou Sanhe Automatic Control Equipment Co., Ltd., Xuzhou, China). Samples were periodically taken for analyses after 1, 3, 5, 7, 10, 14, or 28 days of storage, depending on the studied indicators.

In order to investigate the influence of OHE on the quality, safety, and chemical composition of the pâté, the following measurements were carried out: TAC and thiobarbituric acid reactive substances (TBARS) (0, 3, 5, 7, and 14 days of storage), and physicochemical and microbiological characteristics (0, 3, 7, 10, 14, and 28 days of storage). Proximal and UPLC-ESI-Q-TOF-MS analyses were performed on day 0 of storage.

### 2.4. Proximal Analysis

The techniques employed included the Kjeldahl method for protein, the Soxhlet method with acid hydrolysis for fat, and drying- and vacuum-oven methods for moisture and ash assessment based on the Association of Official Analytical Chemists (AOAC): Official Methods of Analysis [35]; total carbohydrates were calculated by their difference.

### 2.5. Extraction of Meat Pâtés

To determine the TAC and perform UPLC-ESI-Q-TOF-MS analysis, ethanolic extracts of the meat pâtés were prepared, and phosphate extracts were prepared to measure the TBARS. The sample was mixed with 96% ethanol or 50 mM phosphate buffer (pH 7.0) in a ratio of 1:5 (g:mL) and homogenized using an S10 manual homogenizer (Stegler, Guangzhou, China) for 2 min at 9000 rpm. Phosphate extracts were centrifuged at 7000× *g* for 5 min at 4 °C in a 5427R centrifuge (Eppendorf AG, Hamburg, Germany); ethanolic extracts were infused for 60 min at 22 ± 2 °C, followed by filtration through a paper filter. The obtained extracts were stored at minus 40 °C.

### 2.6. UPLC-ESI-Q-TOF-MS Analysis

The metabolome analysis of meat pâtés and OHE was performed using an UHPLC 1290 Infinity system (Agilent Technologies, Santa Clara, CA, USA), as described previously [36], with some modifications. Analysis was performed using a Luna Omega C18 analytical column (2.1 mm × 50 mm, 1.6 µm particle size, Phenomenex Inc., Torrance, CA, USA). The column temperature was maintained at 60 °C, the injection volume was 5 µL, and the linear gradient was as follows: 0% solvent B for 2 min, progression from 0% to 85% solvent B for 8 min, and 85% solvent B for 2 min. The total analysis time was 15 min. An Agilent 6545XT AdvanceBio LC/QTOF (Agilent Technologies, Santa Clara, CA, USA), set to positive ionization mode, was used for the high-pressure ion funnel. The capillary voltage was 4500 V; the nozzle voltage was 2000 V; the drying gas flow was operated at 8 L/min and 325 °C; the gas flow through the casing was operated at 12 L/min and 275 °C; and the atomizer pressure was 30 psi, with a high frequency (RF) of 175 V.

Detected compounds were identified by MS fragmentation using MSDIAL software (ver. 5.1.221218, RIKEN CSRS, Yokohama City, Japan) [37]. The total score for manually selected compounds was ≥80%. Flavonoid contents were determined according to a standard curve using quercetin (Q) in the concentration range of 1–1000 ng/mL [32] and expressed in µg-eq. Q/100 mL OHE or µg-eq. Q/100 g meat pâté.

### 2.7. Stability Assessment

TAC and TBARS were measured after 0, 3, 5, 7, and 14 days of storage at 4 °C; peroxide value (PV), pH, and microbiological parameters were determined after 3, 7, 10, 14, and 28 days of storage at 4 °C.

#### 2.7.1. Ferric Reducing Antioxidant Power (FRAP) Assay

The TAC was measured by the FRAP method using an SF-2000 spectrophotometer (OCB Spectr, St. Petersburg, Russia) according to [38], with some modifications [33]. In brief, 1.45 mL of FRAP reagent and 50 μL of the sample/standard/distilled water (control) were mixed and incubated for 30 min at 37 °C in the dark. The optical density was determined at 594 nm. The standard curve of quercetin (Q) in the concentration range of 140–300 μM was used. The results are expressed in mmol-equiv. Q/L OHE or µmol-equiv. Q/100 g meat pâté.

#### 2.7.2. Lipid Peroxidation Products

The TBARS in the phosphate extracts of the meat pâtés were measured using an SF-2000 spectrophotometer following the method of Brazhe et al. [39], with some modifications [33]. In brief, glass tubes were filled with 1.5 mL of 2% (*w*/*v*) ortho-phosphoric acid, 100 µL of extract or distilled water for the control sample, and 0.5 mL of 0.8% (*w*/*v*) thiobarbituric acid. After incubation at 95 °C for 45 min, samples were cooled to room temperature; then, 2.5 mL of n-butanol was added, and the samples were mixed and centrifuged. Optical density was measured at wavelengths of 535 and 570 nm. TBARS were calculated using the molar extinction coefficient of the (malondialdehyde) MDA-TBA complex (1.56 × 10^5^ M^−1^ cm^−1^) and expressed in µmol/100 g meat pâté.

#### 2.7.3. Determination of Peroxide Value and pH

PV was determined according to the ISO 3960:2017 standard [40] based on the reaction of fat oxidation products (peroxides and hydroperoxides) with potassium iodide in an acidic medium. A solution of sodium thiosulfate was used for titration following the quantitative determination of the released iodine. The results are expressed as mmol. active O_2_/kg of fat. The pH was measured according to the ISO 2917:1999 standard [41] using a FiveEasy FP20 (Mettler Toledo, Stockholm, Sweden).

#### 2.7.4. Microbiological Analyses

The following microbial parameters were determined: total mesophilic aerobic bacteria (TMAB), according to the ISO 4833-1:2013 standard [42]; sulfite-reducing bacteria growing under anaerobic conditions, according to the ISO 15213:2003 standard [43]; yeasts and molds, according to the ISO 21527-2:2008 standard [44]; *Escherichia coli*, according to the ISO 16654:2001 standard [45]; coliform bacteria, according to the ISO 4832:2006 standard [46]; *Salmonella* spp., according to the ISO 6579:2002 standard [47]; *Bacillus cereus*, according to the ISO 7932:2004 standard [48]; presumptive *Pseudomonas* spp., according to the ISO 13720:2010 standard [49]; coagulase-positive staphylococci (*Staphylococcus aureus* and other species), according to the ISO 6888-1:2021 standard [50]; and *Listeria monocytogenes* and *Listeria* spp., according to the ISO 11290-1:2017 standard [51].

### 2.8. Statistical Analyses

The measurements were carried out in triplicate. STATISTICA 17.0 software was used for the statistical analysis. The results were calculated as mean ± SD. Significant differences were tested by non-parametric Mann–Whitney U tests for independent variables; Freidman ANOVAs (n > 2) were used for dependent variables. Differences with *p*-values < 0.10 and 0.05, respectively, were considered statistically significant. After processing the UPLC-ESI-Q-TOF-MS data using the MS-DIAL program (version 5.1.221218, RIKEN CSRS, Yokohama, Japan), the metabolomic peaks were identified, including the collection of peaks, deconvolution, the identification of compounds, and the alignment of peaks to a reference database [37].

## 3. Results

### 3.1. Composition of Meat Pâtés

The physico-chemical composition of the meat pâtés is presented in Table 2.

E1 was characterized by a higher content of protein and ash, statistically exceeding the control indicators by 1.32-fold and 1.08-fold (*p* < 0.10), respectively, while the content of fat and moisture was lower by 1.33% and 4.17% (*p* < 0.10), respectively. E2 demonstrated the same tendency according to the proximal analysis, but the difference between E2 and the control was not as substantial. A decrease in the fat and moisture content accompanied an elevation in the protein content as follows: control > E2 > E1.

### 3.2. Identification of Active Compounds and Metabolome Profile in Meat Pâtés

Ethanolic extracts of the meat pâtés and OHE were investigated by UPLC-ESI-Q-TOF-MS analysis. More than 100 compounds were obtained using the MSDIAL accurate mass tolerance MS1 (0.01 Da) and MS2 (0.05 Da) program parameters. Table S1 (Supplementary Materials) shows the mass parameters and identification characteristics of all manually selected compounds for all samples. A total of 69 compounds were manually selected, including phosphoethanolamines (n = 4); other lipids (n = 10); acyl carnitines (n = 5); alpha amino acids and derivatives (n = 5); B vitamins and related compounds (n = 5); benzodioxoles (n = 5); flavonoids (n = 10); trihydroxy bile acids, alcohols, and derivatives (n = 4); and other organic and polyphenolic compounds (n = 21).

Table 3 shows the main tentative compounds determined in the meat pâtés and OHE; the chromatograms and spectra of the quercetin in the studied samples are presented in Figure S1 (Supplementary Materials); and the ID/structure, representative mass spectra compared to the reference, and total score for each compound are presented in Table S2 (Supplementary Materials). The main flavonoids were quantitatively determined using the calibration curve of quercetin; the regression coefficient was >0.990.

The predicted values of the compounds in the E1 and E2 meat pâtés were calculated based on the OHE results and the volumes added to the recipes for E1 and E2 (Table 1), and they were expected to correspond to ∆ (E1-C) and ∆ (E2-C). Regular spices were added to the recipes of both the control and experimental meat pâtés, which were also a source of antioxidants; therefore, the values of the control sample were subtracted from the experimental values.

The total amount of flavonoids in E1 and E2 was significantly higher than in the control by 2.8-fold (*p* < 0.1) and 1.9-fold (*p* < 0.1), respectively, while the difference between E1 and E2 averaged 1.5-fold (*p* < 0.1). The content of all flavonoids in E1 and E2 exceeded the control values, except for petunidin 3-galactoside. Thus, the concentrations of delphinidin 3-galactoside, luteolin-4′-O-glucoside, spiraeoside, myricitrin, isorhamnetin-3-O-beta-D-Glucoside, isorhamnetin, kaempferol, and quercetin in E1 were greater than in the control by 4.3-fold (*p* < 0.1), 2.0-fold (*p* < 0.1), 1.9-fold (*p* < 0.1), 2.6-fold (*p* < 0.1), 1.4-fold (*p* < 0.1), 6.0-fold (*p* < 0.1), 18.0-fold (*p* < 0.1), and 131.9-fold (*p* < 0.1), respectively, while for E1 these differences were 1.9-fold (*p* < 0.1), 1.4-fold (*p* < 0.1), 1.5-fold (*p* < 0.1), 2.0-fold (*p* < 0.1), 1.5-fold (*p* < 0.1), 3.3-fold (*p* < 0.1), 7.4-fold (*p* < 0.1), and 58.5-fold (*p* < 0.1), respectively. Quercetin 3-O-malonylglucoside, quercetin-3,4′-O-di-beta-glucoside, and baimaside presented in trace amounts in the control, while the content of these compounds in E1 and E2 was in the range of 8.37–73.66 µg-eq. Q/100 g meat pâté. The content of all flavonoids in E1, except for petunidin 3-galactoside and isorhamnetin-3-O-beta-D-Glucoside, exceeded the E2 values by 1.3–2.4-fold (*p* < 0.1).

Based on the determination of the main compounds in OHE, the predicted content in E1, E2, ∆ (E1-C), and ∆ (E2-C) was calculated in order to evaluate the antioxidant stability of *Allium cepa* husk components during meat pâté processing. We found that the content in ∆ (E1-C) and ∆ (E2-C) was lower than predicted. However, the difference between ∆ (E1-C) and ∆ (E2-C) averaged 1.9-fold, which corresponded to the ratio of OHE volume in E1 and E2.

### 3.3. Determination of Antioxidant Stability

The results of the TAC determination for the meat pâtés using the FRAP method after 0, 3, 5, 7, and 14 days of storage at 4 °C are presented in Table 4.

During storage, a decrease in the TAC_FRAP_ was observed in all samples. The TAC_FRAP_ of E1 and E2 for all storage durations statistically exceeded the indicators for the control meat pâté. Thus, the TAC_FRAP_ of E1, with an ethanolic OHE volume of 68 µL/g of raw materials, exceeded that of the control pâté by 36.67 ± 5.86 µmol-equiv. Q/100 g meat pâté, whereas the TAC_FRAP_ of E2, with an OHE volume of 34 µL/g exceeded the control value by 16.39 ± 2.75 µmol-equiv. Q/100 g meat pâté. We determined that a 50% decrease in the volume of OHE in the meat product recipe led to a statistically significant reduction in the contribution of the plant extract to the TAC_FRAP_. On day 0, the difference between the TAC_FRAP_ of E1 and E2, as well as the ∆ values, was about 2.3 times.

The decreases in the TAC_FRAP_ of the OHE during meat product processing were calculated and are presented in Table 5.

When more OHE was added to the recipe, greater decreases in the TAC_FRAP_ were observed. Thus, the decrease for E1 averaged 17.41%, while it averaged only 9.45% for E2.

### 3.4. Determination of Storage Indicators

The results of the PV, pH, TBARS, and microbiological parameter determination are presented in Table 6, Table 7 and Table 8.

The PV of all samples increased by statistically significant amounts over the 28 days of storage (*p* < 0.05) but did not change significantly during the first 7 days (*p* > 0.05). The PV of E2 was the smallest on day 0, being significantly lower than that of the control and E1 by 0.72 and 0.74 mmol. active O_2_/kg of fat (*p* < 0.10), respectively. However, on day 3 of storage, E2 achieved the highest PV, which was 0.57 mmol. active O_2_/kg of fat (*p* < 0.10) higher than that of the control but did not differ from that of E1. After 7 days of storage, the PV of E2 was statistically lower than that of the control and E1, and after 10 days it was higher than that of E1 but lower than that of the control.

The pH value changed by statistically significant amounts in the control and E1 over the 28 days of storage (*p* < 0.05) but did not change significantly in any sample during the first 7 days of storage. On day 0 and 3 of storage, the pH of E1 did not differ from that of the control, while the pH of E2 did not differ from that of the control on day 0 alone. On day 0, the pH of E2 was lower than that of E1 and the control, but after 3 days of storage E2 demonstrated the highest pH value, which statistically differed from that of the control and E1. Despite the observed differences, the pH of the meat pâtés varied in the range of 5.83–6.07, representing an insignificant change for this type of product.

The TBARS of the meat pâtés did not differ significantly on day 0. However, the TBARS in the control were slightly increased by about 0.25 µmol/100 g meat pâté. After 3, 5, 7, and 14 days of storage, we found a significant difference in the TBARS between the control and experimental samples, while we observed no statistical difference between the TBARS of E1 and E2. On day 3 of storage, the concentration of TBARS in the control exceeded that in E1 and E2 by 2.42-fold and 2.1-fold (*p* < 0.10), respectively. During storage, a statistical change in TBARS was observed for the control and E2 (*p* < 0.05), whereas the TBARS in E1 changed only slightly. The TBARS in E1 remained unchanged for 5 days, while those in E2 remained unchanged for 3 days.

Table S3 (Supplementary Materials) shows the results of the microbiological analyses of the meat pâtés during storage at 4 °C, showing that the developed meat pâtés were safe according to all indicators of microbial spoilage within 10 days of production.

## 4. Discussion

Recently, a trend of replacing synthetic antioxidants with natural ones has emerged, including for the purpose of enriching foods with essential nutrients [52]. Antioxidants from natural sources are a good alternative to synthetic antioxidants due to their high content of phenols and other active components that can effectively prevent lipid oxidation [24]. Plant extracts are becoming important additives in the food industry due to their antimicrobial and antioxidant properties, which delay the development of undesirable tastes and improve the color stability of meat products [21]. Therefore, the use of antioxidants in the meat industry is a reasonable and necessary step to extend the shelf lives and maintain the organoleptic and nutritional qualities of meat products. From this point of view, the use of plant extracts in the food industry contributes to improving the functionality of meat products, developing products for a healthy lifestyle, and manufacturing clean-label foods without or with a minimal content of synthetic additives [21,53,54,55].

Numerous studies have shown the effectiveness of using plant extracts or ingredients to slow down oxidative processes in meat products. Monica Gallo et al. demonstrated the effectiveness of *Echinacea angustifolia* extract for reducing the oxidation of lipids and proteins in chicken meat [16]. Mario Estévez et al. slowed down the lipid peroxidation in liver pâté through the addition of sage and rosemary essential oils [11]. Lilian Regina Barros Mariutti et al. demonstrated that the addition of sage to chicken meat (0.1 g/100 g) is a good alternative to prevent and slow down the formation of compounds as a result of lipid oxidation that are responsible for unpleasant tastes and a loss of nutritional qualities during prolonged freezer storage [56]. Pellegrini et al. assessed the effect of partial fat replacement with quinoa paste on the quality of pork liver pâté [9]. The authors found that replacing 10% of the fat lowered the oxidation rate of the product in comparison to that of the control sample. An equally promising source of plant antioxidants is the waste from the fruit and vegetable industry. This area is currently under study, and biologically active substances obtained from such waste have been used as antioxidants. Jose M. Lorenzo et al. demonstrated the possibilities of using peanut skins, which are a by-product of peanut production, as a source of antioxidant compounds, especially proanthocyanidins, which are capable of inhibiting oxidative reactions involving mainly pigments, lipids, and proteins [57]. Our results demonstrated that the addition of yellow onion husk ethanolic extract to meat pâté at a volume of 68 µL/g or 34 µL/g unsalted blanched materials to replace 34% or 17% of the beef broth, respectively, corresponding to a TAC_FRAP_ value of 44.4 or 22.2 µmol-equiv. Q/100 g meat pâté, respectively, (i.e., 13.42 or 6.71 mg of quercetin/100 g meat pâté) allowed us to maintain an increased content of antioxidants, which decreased the generation of secondary products of lipid peroxidation for 14 days at 4 °C. However, the TAC_FRAP_ of both the control and experimental samples declined during storage, which was explained by the inhibition of oxidation products by antioxidants from the spices and OHE. This observation was confirmed by the increases in the PV and TBARS during storage. Remarkably, the increases in the lipid oxidation indicators in the experimental samples were not as noticeable as those in the control samples. We also showed that the addition of 34 µL/g of the extract to the recipe better preserved the effectiveness of the antioxidants. Thus, for their entire shelf life, a TAC decrease of 33.93% was noted for the control sample, 31.27% for E1, and only 27.60% for E2.

The control meat pâté also demonstrated a prominent TAC_FRAP_ and, according to the results of the UPLC-ESI-Q-TOF-MS analysis, contained almost all the detected flavonoids. Regular spices were included in the recipes for both the control and experimental meat pâtés, such as black pepper, allspice, mustard, and nutmeg, which are also sources of antioxidants. Black pepper contains flavonoids such as catechin, quercetin, myricetin, carotenoids [58], kaempferol 3-O-glucosyl-rhamnosyl-galactoside myricetin 3-O-rhamnoside, kaempferol 3-O-(2″-rhamnosyl-galactoside) 7-O-rhamnoside, isorhamnetin 3-O-glucoside7-O-rhamnoside, and delphinidin 3-O-glucosyl-glucoside [59], and its content of total flavonoids could reach 2149 μg QE/100 g [60]. Allspice also contains flavonoids, such as quercetin [61], gallic acid, quercetin 3-O-β-D-galactoside, quercetin 3-O-β-D-glucuronide 6″-methyl ester, myricetin, myricetin 3-O-β-D-galactoside, kaempferol 3-O-β-D-glucoside, and kaempferol 3-O-β-D-galactoside, which were isolated by repeated column chromatography from dried ground berries of allspice in [62]. A total of 26 compounds (phenolic acids, flavonoids, and glucosinolates) were identified in mustard samples [63], and among the flavonoids, the most abundant were quercetin and epicatechin [64]. Flavones, flavonols, and anthocyanidins appear to be more abundant in nutmeg [65]. Nutmeg could contain saponin, alkaloid, tannin, flavonoids, α-pinene, β-pinene, myrcene, 1,8-cineole, carvacrol, terpinen-4-ol, sabinene, camphene, myristicin, elemicin, isoelemicin, eugenol, etc. [66,67]. Although the presence of almost all of the detected flavonoids in the control meat pâté could be explained by the regular spices included in the recipe, surprisingly, spiraeoside was the most abundant antioxidant in this sample, averaging 11,041.1 ± 158.1 µg-eq. Q/100 g meat pâté. Spiraeoside (quercetin 3,4′-diglucoside) is one of the predominant flavonoids present in the husk of *Allium cepa* L. [68], alongside quercetin (the most abundant in onion husks) and its glycosides, kaempferol and myricetin [32,69,70,71,72]. However, these are not abundant in black pepper, allspice, mustard, or nutmeg. Fried onions were included in the meat pâté recipes. Despite our previous results showing that the total antioxidant capacity of onion bulbs is much lower than that of onion husks [73], as well as data reporting that the quercetin content in onion husks is about 32 times higher than in the edible parts of onions [70], quercetin 3,4′-O-diglucoside was detected as a major flavonol in onion bulbs [74], and yellow onion bulbs tend to accumulate quercertin-3,4′-diglucoside [75]. However, in this study we demonstrated that the flavonoid content in the experimental meat pâtés was significantly higher than in the control samples. Moreover, the addition of onion husk extract in both volumes during meat pâté processing ensured the presence of significant amounts of quercetin, the main flavonoid in onion husks, in developed products in appropriate ratio.

## 5. Conclusions

We found that the introduction of ethanolic yellow onion husk extract into the meat matrix at a volume of 68 µL/g of raw materials reduced the indicators of oxidative spoilage and increased the total antioxidant capacity by more than 1.5 times, which was maintained for 14 days of storage at 4 °C. A reduction in the amount of added ethanolic yellow onion husk extract ensured the preservation of the antioxidant effect in the meat matrix for 14 days of storage, allowing a decrease in the concentration of ethanol in the developed meat pâté. Our results support the use of yellow onion husk extract in the food industry to contribute to improving the functionality of meat products, developing products for a healthy lifestyle, and providing clean-label foods without or with a minimal content of synthetic additives.

## Figures and Tables

**Table 1:** Recipes for meat pâtés.

Ingredient	Control	E1 ^#^	E2 ^#^
Blanched materials, g/kg blanched materials			
Beef flank	350	350	350
Beef liver	230	230	230
Lean pork	200	200	200
Pork heart	100	100	100
Wheat flour	50	50	50
Powdered cow’s milk	20	20	20
Fried onions	50	50	50
Auxiliary materials, g/kg unsalted blanched materials			
Table salt	14	14	14
Sugar	3	3	3
Ground black pepper	1	1	1
Ground allspice	0.5	0.5	0.5
Ground mustard	5	5	5
Nutmeg	0.5	0.5	0.5
Auxiliary materials, mL/kg unsalted blanched materials			
Beef broth after blanching	200	132	166
OHE *	-	68	34

* OHE—yellow onion (*Állium cépa*) husk ethanolic extract; **^#^** OHE was added instead of broth at volumes of 68 µL/g for experiment 1 (E1) and 34 µL/g for experiment 2 (E2).

**Table 2:** Proximal analysis of meat pâtés.

	Fat, %	Protein, %	Moisture, %	Ash, %	Carbohydrates, %
Control	9.33 ± 0.06	21.27 ± 0.06	57.17 ± 0.21	1.95 ± 0.04	10.29 ± 0.12
E1	8.00 ± 0.20 *	28.03 ± 0.15 *	53.0 ± 0.17 *	2.11 ± 0.06 *	8.85 ± 0.43 *
E2	8.93 ± 0.15 *^,#^	24.0 ± 0.10 *^,#^	55.7 ± 0.20 *^,#^	2.10 ± 0.06 *	9.27 ± 0.34 *

* Significant difference as compared to control (based on two-tailed Mann–Whitney U test, *p* < 0.1); ^#^ significant difference between E2 and E1 (based on two-tailed Mann–Whitney U test, *p* < 0.1).

**Table 3:** Compounds in meat pâtés and OHE.

Metabolite	OHE, µg-eq. Q/100 mL	µg-eq. Q/100 g Meat Pâté						
		Predicted (Calculated)		Control (C)	Obtained			
		E1	E2		E1	E2	∆ (E1-C)	∆ (E2-C)
Delphinidin 3-galactoside	955.5 ± 59.4	65.0 ± 4.0	32.5 ± 2.0	18.8 ± 2.6	80.4 ± 34.2 *	35.7 ± 3.1 *^,#^	61.6	17.0
Petunidin 3-galactoside	293.5 ± 19.8	20.0 ± 1.4	10.0 ± 0.7	13.1 ± 4.6	15.6 ± 3.5	19.1 ± 5.9	2.5	6.0
Luteolin-4′- O-glucoside	804.9 ± 56.9	53.7 ± 3.9	27.4 ± 1.9	30.9 ± 2.2	61.8 ± 8.2 *	43.3 ± 8.9 *	30.9	12.5
Spiraeoside	365,200.0 ± 0.0	24,833.6 ± 0.0	12,416.8 ± 0.0	11,041.1 ± 158.1	20,809.7 ± 372.8 *	16,447.7 ± 504.1 *^,#^	9768.6	5406.7
Myricitrin	6534.4 ± 344.7	444.3 ± 23.4	222.2 ± 11.7	254.9 ± 20.0	656.7 ± 8.1 *	506.9 ± 32.4 *^,#^	402.1	252.0
Isorhamnetin-3-O-beta-D- Glucoside	2789.6 ± 53.1	189.7 ± 3.6	94.9 ± 1.8	180.5 ± 25.8	259.0 ± 6.3 *	273.4 ± 21.0 *	78.4	92.8
Quercetin 3-O-malonylglucoside	441.0 ± 17.5	30.0 ± 1.2	15.0 ± 0.6	0.0 ± 0.0	15.0 ± 6.4 *	8.4 ± 1.7 *	15.0	8.4
Quercetin-3,4′-O-di-beta- glucoside	1796.9 ± 121.3	122.2 ± 8.3	61.1 ± 4.1	0.1 ± 0.2	73.7 ± 3.8 *	40.0 ± 9.2 *^,#^	73.5	39.9
Baimaside	449.7 ± 34.0	30.6 ± 2.3	15.3 ± 1.2	0.4 ± 0.8	19.4 ± 10.2 *	11.4 ± 0.3 *	19.0	11.0
Isorhamnetin	1831.1 ± 132.9	124.5 ± 9.0	62.3 ± 4.5	24.2 ± 6.7	144.0 ± 7.7 *	80.1 ± 11.3 *^,#^	119.8	55.9
Kaempferol	1057.3 ± 29.1	71.9 ± 2.0	36.0 ± 1.0	3.0 ± 1.9	53.8 ± 10.4 *	22.1 ± 1.8 *^,#^	50.8	19.1
Quercetin	174,546.7 ± 3596.7	11,869.2 ± 244.6	5934.6 ± 122.3	77.9 ± 10.1	10,279.0 * ± 389.8 *	4563.6 * ± 76.8 *^,#^	10,201.0	4485.7
Total	556,700.5 ± 3495.1	37,855.6 ± 237.8	18,927.8 ± 118.8	11,644.9 ± 164.3	32,468.2 * ± 664.1 *	21,949.4 ± 417.5 *^,#^	20,823.3	10,304.5

* Significant difference as compared to control (based on two-tailed Mann–Whitney U test, *p* < 0.1); ^#^ significant difference between E2 and E1 (based on two-tailed Mann–Whitney U test, *p* < 0.1).

**Table 4:** TAC of meat pâtés during storage at 4 °C.

Day	TAC_FRAP_, µmol-equiv. Q/ 100 g Meat Pâté				
	Control (C)	E1	E2	∆ (E1-C)	∆ (E2-C)
0	32.83 ± 1.19	72.94 ± 1.85 *	50.52 ± 1.27 *^,#^	40.11	17.69
3	29.96 ± 0.79	73.35 ± 0.30 *	49.88 ± 0.67 *^,#^	43.39	19.92
5	26.19 ± 1.21	64.25 ± 1.03 *	42.97 ± 0.20 *^,#^	38.06	16.78
7	25.06 ± 0.20	58.42 ± 0.29 *	37.73 ± 0.29 *^,#^	33.36	12.67
14	21.69 ± 0.44	50.13 ± 0.97 *	36.59 ± 0.14 *^,#^	28.44	14.90

* Significant difference as compared to control (based on two-tailed Mann–Whitney U test, *p* < 0.1); ^#^ significant difference between E2 and E1 (based on two-tailed Mann–Whitney U test, *p* < 0.1).

**Table 5:** Decreases in TAC_FRAP_ during meat product processing.

µmol-equiv. Q/100 g Meat Pâté		Decrease in TAC_FRAP_ in E1, %	µmol-equiv. Q/100 g Meat Pâté		Decrease in TAC_FRAP_ in E2, %
Amount in Recipe E1	∆ (E1-C)		Amount in Recipe E2	∆ (E2-C)	
44.4	36.67 ± 5.86	17.41	22.2	16.39 ± 2.75	9.45

**Table 6:** PV of meat pâtés during storage at 4 °C.

Day	PV, mmol. Active O_2_/kg of Fat		
	Control	E1	E2
0	2.07 ± 0.34	2.09 ± 0.16	1.35 ± 0.25 *^,#^
3	2.0 ± 0.37	2.44 ± 0.12 *	2.57 ± 0.14 *
7	2.83 ± 0.04	2.98 ± 0.14	2.61 ± 0.05 *^,#^
10	3.62 ± 0.09	3.32 ± 0.03 *	3.54 ± 0.06 ^#^
14	3.65 ± 0.16	3.84 ± 0.22	4.07 ± 0.02 *
28	4.19 ± 0.11	3.92 ± 0.23	3.96 ± 0.23
*p*-value ^1^ (0–28 days)	0.014	0.012	0.014
*p*-value ^1^ (0–7 days)	0.097	0.050	0.097

* Significant difference as compared to control (based on two-tailed Mann–Whitney U test, *p* < 0.1); ^#^ significant difference between E2 and E1 (based on two-tailed Mann–Whitney U test, *p* < 0.1); ^1^ based on a Freidman ANOVA, *p* < 0.05.

**Table 7:** pH of meat pâtés during storage at 4 °C.

Day	pH Value		
	Control	E1	E2
0	6.07 ± 0.06	6.09 ± 0.04	5.98 ± 0.05 ^#^
3	5.95 ± 0.03	5.92 ± 0.03	6.05 ± 0.04 *^,#^
7	5.95 ± 0.02	5.83 ± 0.03 *	6.02 ± 0.04 *^,#^
10	5.89 ± 0.03	5.83 ± 0.02 *	6.00 ± 0.01 *^,#^
14	5.93 ± 0.04	5.87 ± 0.02 *	6.02 ± 0.01 *^,#^
28	6.0 ± 0.05	5.91 ± 0.02 *	6.06 ± 0.03 ^#^
*p*-value ^1^ (0–28 days)	0.025	0.014	0.187
*p*-value ^1^ (0–7 days)	0.097	0.050	0.202

* Significant difference as compared to control (based on two-tailed Mann–Whitney U test, *p* < 0.1); ^#^ significant difference between E2 and E1 (based on two-tailed Mann–Whitney U test, *p* < 0.1); ^1^ based on a Freidman ANOVA, *p* < 0.05.

**Table 8:** TBARS of meat pâtés during storage at 4 °C.

Day	TBARS, µmol/100 g Meat Pâté		
	Control	E1	E2
0	2.27 ± 0.30	1.96 ± 0.19	1.94 ± 0.36
3	4.57 ± 0.32	1.89 ± 0.33 *	2.20 ± 0.35 *
7	5.83 ± 3.83	2.11 ± 0.13 *	2.96 ± 0.73 *
10	6.07 ± 0.17	2.80 ± 0.57 *	2.26 ± 0.34 *
14	4.79 ± 0.73	2.29 ± 0.69 *	2.83 ± 0.53 *
*p*-value ^1^ (0–14 days)	0.009	0.114	0.027

* Significant difference as compared to control (based on two-tailed Mann–Whitney U test, *p* < 0.1); ^1^ based on a Freidman ANOVA, *p* < 0.05.

## Data Availability

Data are contained within the article or Supplementary Materials.

## Supplementary Materials

The following supporting information can be downloaded at: https://www.mdpi.com/article/10.3390/antiox12051103/s1, Table S1: Mass parameters and identification characteristics of all manually selected compounds, Figure S1: Chromatogram and spectrum of quercetin in the studied samples, Table S2: Results of microbiological analyses of meat pâtés during storage at 4 °C. Table S3. Results of microbiological analyses of meat pâtés during storage at 4 °C.
