# Supplementary material for: Assessment of Antioxidant Stability of Meat Pâté with Allium cepa Husk Extract
Source: Antioxidants (Basel). 2023 May 16;12(5):1103. doi: 10.3390/antiox12051103 (PMC10215231; doi:10.3390/antiox12051103)
Supplement: Supplementary file 1 [file antioxidants-12-01103-s001.zip › antioxidants-2312772-supplementary.docx]

**Table S1.** Mass parameters and identification characteristics of all manually selected compounds

| **Metabolite** | **Adduct type** | **Ontology** | **Total score** |
| --- | --- | --- | --- |
| PC(16:0/0:0) | [M+H]+ | 1-acyl-sn-glycero-3-phosphocholines | 0.83 |
| 1-heptadecanoyl-2-hydroxy-sn-glycero-3-phosphocholine | [M+H]+ | 1-acyl-sn-glycero-3-phosphocholines | 0.85 |
| 1-Stearoyl-sn-glycero-3-phosphocholine | [M+H]+ | 1-acyl-sn-glycero-3-phosphocholines | 0.89 |
| 1-palmitoyl-2-hydroxy-sn-glycero-3-phosphoethanolamine | [M+H]+ | 1-acyl-sn-glycero-3-phosphoethanolamines | 0.94 |
| Monoolein | [M+H]+ | 1-monoacylglycerols | 0.91 |
| LysoPC(0:0/18:0) | [M+Na]+ | 2-acyl-sn-glycero-3-phosphocholines | 0.84 |
| Histamine | [M+H]+ | 2-arylethylamines | 0.82 |
| Adenine hydrochloride | [M+H]+ | 6-aminopurines | 0.82 |
| 4-[5-(4-hydroxy-3-methoxyphenyl)-3,4-dimethyloxolan-2-yl]-2-methoxyphenol | [M+H]+ | 7,7'-epoxylignans | 0.88 |
| Hexanoyl-L-Carnitine | [M+H]+ | Acyl carnitines | 0.88 |
| Octanoyl-L-Carnitine | [M+H]+ | Acyl carnitines | 0.89 |
| Decanoyl-L-Carnitine | [M+H]+ | Acyl carnitines | 0.83 |
| Lauroylcarnitine | [M+H]+ | Acyl carnitines | 0.89 |
| Palmitoylcarnitine | [M+H]+ | Acyl carnitines | 0.91 |
| BETAINE | [M+H]+ | Alpha amino acids | 0.94 |
| Creatine | [M+H]+ | Alpha amino acids and derivatives | 0.82 |
| Cyclo(Leu-Pro) | [M+H]+ | Alpha amino acids and derivatives | 0.82 |
| (E)-5-hydroxy-3-isobutyl-6-(3-methylbenzylidene)-1,6-dihydropyrazin-2(3H)-one | [M+H]+ | Alpha amino acids and derivatives | 0.75 |
| Delphinidin 3-galactoside | [M]+ | Anthocyanidin-3-O-glycosides | 0.84 |
| Petunidin 3-galactoside | [M]+ | Anthocyanidin-3-O-glycosides | 0.95 |
| Piperyline | [M+H]+ | Benzodioxoles | 0.87 |
| Piperanine | [M+H]+ | Benzodioxoles | 1 |
| Pipernonaline | [M+H]+ | Benzodioxoles | 0.8 |
| Piperolein B | [M+H]+ | Benzodioxoles | 0.92 |
| Guineensine | [M+H]+ | Benzodioxoles | 0.8 |
| L-Carnitine | [M+H]+ | Carnitines | 1 |
| Ceramide (18:1/16:0) | [M+H]+ | Ceramides | 0.94 |
| Sinapine | [M+H]+ | Coumaric acids and derivatives | 0.92 |
| Lathyrol | [M+Na]+ | Diterpenoids | 0.77 |
| 7,8-dimethylalloxazine (lumichrome) | [M+H]+ | Flavins | 0.8 |
| Riboflavin | [M+H]+ | Flavins | 0.93 |
| Luteolin-4'-O-glucoside | [M+H]+ | Flavonoid O-glycosides | 0.98 |
| Spiraeoside | [M+H]+ | Flavonoid O-glycosides | 0.98 |
| Myricitrin | [M+Na]+ | Flavonoid-3-O-glycosides | 0.82 |
| Isorhamnetin-3-O-beta-D-Glucoside | [M+Na]+ | Flavonoid-3-O-glycosides | 0.93 |
| Quercetin 3-O-malonylglucoside | [M+H]+ | Flavanonols | 0.86 |
| Quercetin-3,4'-O-di-beta-glucoside | [M+H]+ | Flavonoid-3-O-glycosides | 0.88 |
| Baimaside | [M+Na]+ | Flavonoid-3-O-glycosides | 0.84 |
| Isorhamnetin | [M+H]+ | Flavonol O-glycosides | 0.91 |
| Kaempferol | [M+H]+ | Flavonols | 0.9 |
| Quercetin | [M+H]+ | Flavonols | 0.98 |
| 5-O-methylvisammioside | [M+H]+ | Furanochromones | 0.94 |
| Colchicine derivative | [M+H]+ | Gamma amino acids and derivatives | 0.8 |
| sn-Glycero-3-phosphocholine | [M]+ | Glycerophosphocholines | 0.91 |
| Glycochenodeoxycholate | [M-2H2O+H]+ | Glycinated bile acids and derivatives | 0.83 |
| Glycocholic Acid | [M+H]+ | Glycinated bile acids and derivatives | 0.97 |
| Carnosine | [M+H]+ | Hybrid peptides | 0.93 |
| trans-Ferulic acid | [M+H-H2O]+ | Hydroxycinnamic acids | 0.84 |
| feruloyltyramine | [M+H]+ | Hydroxycinnamic acids and derivatives | 1.01 |
| Hypoxanthine | [M+H]+ | Hypoxanthines | 0.97 |
| Tryptophan | [M+H]+ | Indolyl carboxylic acids and derivatives | 0.91 |
| 4-[2-(2,6-dimethoxy-4-prop-2-enylphenoxy)-1-hydroxypropyl]-2-methoxyphenol | [M+Na]+ | Lignans, neolignans and related compounds | 0.95 |
| 2-(2,6-dimethoxy-4-prop-2-enylphenoxy)-1-(3,4,5-trimethoxyphenyl)propan-1-ol | [M+Na]+ | Lignans, neolignans and related compounds | 0.84 |
| LPC 18:1 | [M+H]+ | Lipids | 0.87 |
| Colchicine derivative | [M+Na]+ | Methionine and derivatives | 0.93 |
| Coniferyl alcohol | [M+Na]+ | Methoxyphenols | 0.85 |
| (2E,4E)-N-(2-methylpropyl)dodeca-2,4-dienamide | [M+H]+ | N-acyl amines | 0.89 |
| (2E,4E,12Z)-N-(2-methylpropyl)octadeca-2,4,12-trienamide | [M+H]+ | N-acyl amines | 0.87 |
| Nicotinamide | [M+H]+ | Nicotinamides | 0.96 |
| Melezitose | [M+Na]+ | Oligosaccharides | 0.93 |
| Aspartame | [M+H]+ | Peptides | 0.79 |
| L-Phenylalanine | [M+H]+ | Phenylalanine and derivatives | 0.93 |
| Benzalkonium chloride (C12) | [M]+ | Phenylmethylamines | 0.85 |
| Phosphocholine | [M+H]+ | Phosphocholines | 0.93 |
| Adenosine | [M+H]+ | Purine nucleosides | 0.88 |
| inosine | [M+Na]+ | Purine nucleosides | 0.84 |
| Cholic Acid | [M+NH4]+ | Trihydroxy bile acids, alcohols and derivatives | 0.89 |
| taurocholic acid | [M+H]+ | Trihydroxy bile acids, alcohols and derivatives | 0.81 |
| Xanthine | [M+H]+ | Xanthines | 0.81 |


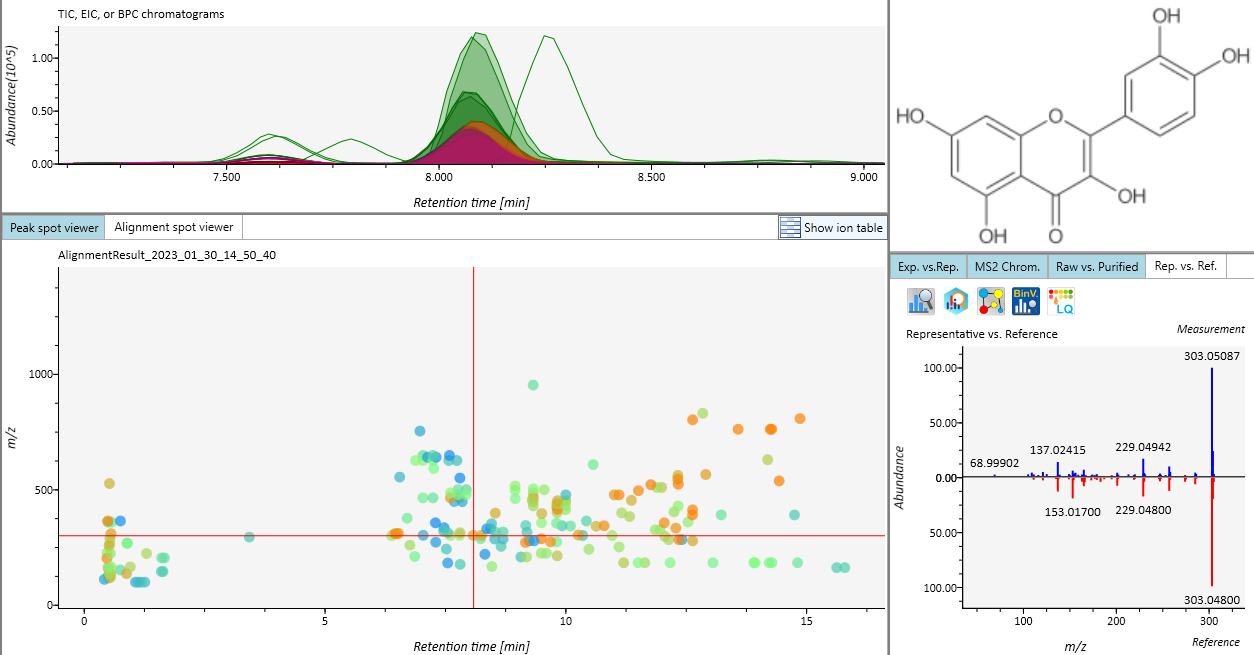


**Figure S1.** Chromatogram and spectrum of quercetin in the studied samples.

**Table S2.** ID/Structure, mass spectra representative vs. reference and total score for compounds.

| **Metabolite** | **ID/Structure** | **Representative vs. Reference** | **Total score** |
| --- | --- | --- | --- |
| Delphinidin  3-galactoside | 4264  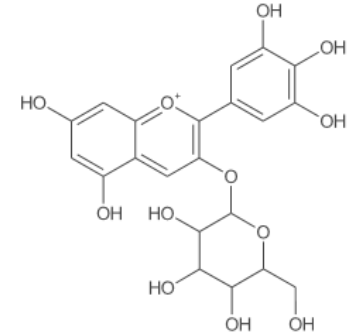 |  | 0.84 |
| Petunidin  3-galactoside | 4425  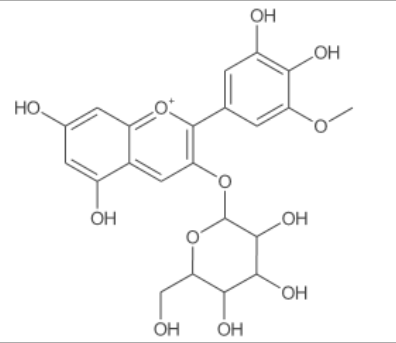 |  | 0.95 |
| Luteolin-4'-  O-glucoside | 4087  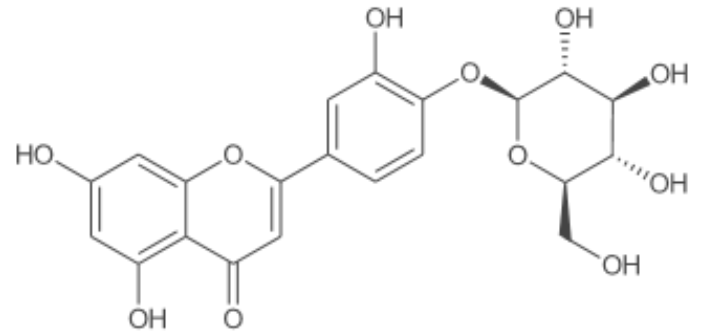 |  | 0.98 |
| Spiraeoside | 4266  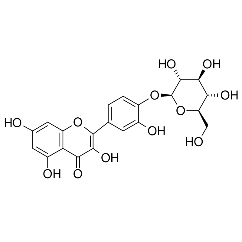 |  | 0.98 |
| Myricitrin | 4536  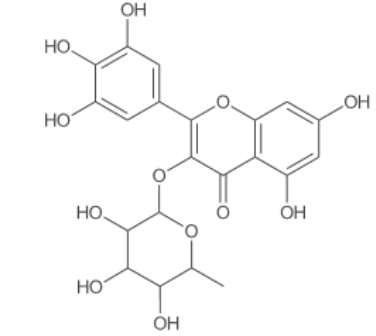 |  | 0.82 |
| Isorhamnetin-3-O-beta-D-  Glucoside | 4708  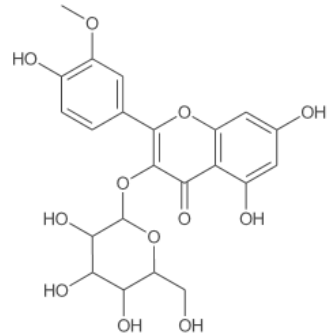 |  | 0.93 |
| Quercetin 3-O-malonylglucoside | 5639  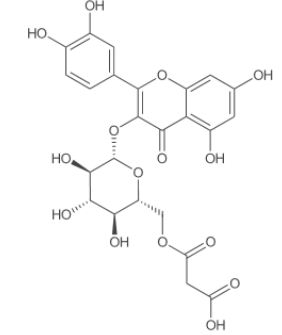 |  | 0.86 |
| Quercetin-3,4'-O-di-beta-  glucoside | 6872  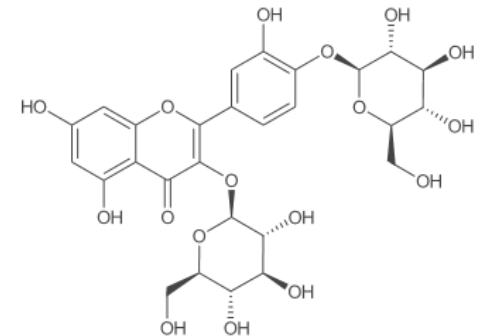 |  | 0.88 |
| Baimaside | 7270  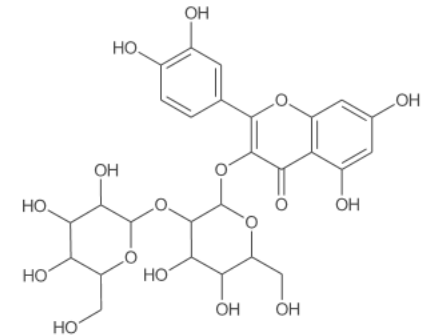 |  | 0.84 |
| Isorhamnetin | 2276  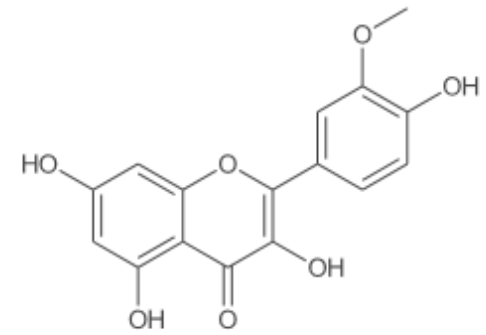 |  | 0.91 |
| Kaempferol | 1922  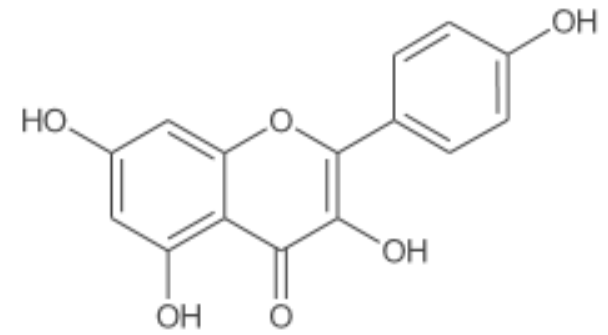 |  | 0.90 |
| Quercetin | 2111  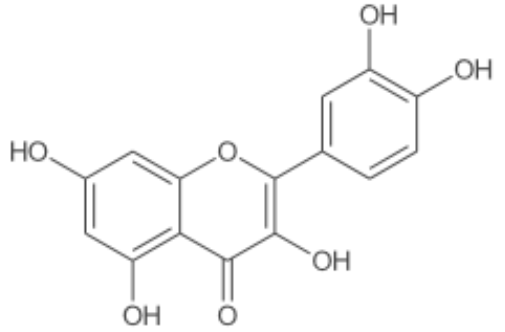 |  | 0.98 |

**Table S3.** Results of microbiological analyses of meat pâtés during storage at 4 °C.

| **Days** | **TMAB, GFU/g** | | | **Yeasts, GFU/g** | | |
| --- | --- | --- | --- | --- | --- | --- |
|  | **Control** | **E1** | **E2** | **Control** | **E1** | **E2** |
| 0 | 1.0x10^1^ | 1.1x10^1^ | 2.0x10^1^ | < 1x10^1^ | < 1x10^1^ | < 1x10^1^ |
| 3 | 1.2x10^2^ | 1.5x10^2^ | 2.5x10^2^ | < 1x10^1^ | < 1x10^1^ | < 1x10^1^ |
| 7 | 5.0x10^2^ | 4.0x10^2^ | 1.0x10^3^ | < 1x10^1^ | < 1x10^1^ | < 1x10^1^ |
| 10 | 2.1x10^2^ | 8.6x10^2^ | 2.1x10^2^ | < 1x10^1^ | < 1x10^1^ | < 1x10^1^ |
| 14 | 1.2x10^3^ | 5.4x10^3^ | 3.7x10^3^ | < 1x10^1^ | < 1x10^1^ | < 1x10^1^ |
|  | **Sulfite-reducing bacteria** | | | **Moulds, GFU/g** | | |
| 0 | N.D. | N.D. | N.D. | N.D. | N.D. | N.D. |
| 3 | N.D. | N.D. | N.D. | N.D. | N.D. | N.D. |
| 7 | N.D. | N.D. | N.D. | < 1x10^1^ | < 1x10^1^ | < 1x10^1^ |
| 10 | N.D. | N.D. | N.D. | N.D. | N.D. | N.D. |
| 14 | N.D. | N.D. | N.D. | < 1x10^1^ | < 1x10^1^ | < 1x10^1^ |
|  | ***E. Coli*** | | | **Coliforms bacteria** | | |
| 0 | N.D. | N.D. | N.D. | N.D. | N.D. | N.D. |
| 3 | N.D. | N.D. | N.D. | N.D. | N.D. | N.D. |
| 7 | N.D. | N.D. | N.D. | N.D. | N.D. | N.D. |
| 10 | N.D. | N.D. | N.D. | N.D. | N.D. | N.D. |
| 14 | N.D. | N.D. | N.D. | N.D. | N.D. | N.D. |
|  | ***Salmonella spp.*** | | | ***B. Cereus*** | | |
| 0 | N.D. | N.D. | N.D. | N.D. | N.D. | N.D. |
| 3 | N.D. | N.D. | N.D. | N.D. | N.D. | N.D. |
| 7 | N.D. | N.D. | N.D. | N.D. | N.D. | N.D. |
| 10 | N.D. | N.D. | N.D. | N.D. | N.D. | N.D. |
| 14 | N.D. | N.D. | N.D. | N.D. | N.D. | N.D. |
|  | ***Pseudomonas spp*** | | | ***S. Aureus*** | | |
|  | N.D. | N.D. | N.D. | N.D. | N.D. | N.D. |
|  | N.D. | N.D. | N.D. | N.D. | N.D. | N.D. |
|  | N.D. | N.D. | N.D. | N.D. | N.D. | N.D. |
|  | N.D. | N.D. | N.D. | N.D. | N.D. | N.D. |
|  | N.D. | N.D. | N.D. | N.D. | N.D. | N.D. |
|  | ***L. monocytogenes*** | | |  | | |
| 0 | N.D. | N.D. | N.D. |  |  |  |
| 3 | N.D. | N.D. | N.D. |  |  |  |
| 7 | N.D. | N.D. | N.D. |  |  |  |
| 10 | N.D. | N.D. | N.D. |  |  |  |
| 14 | N.D. | N.D. | N.D. |  |  |  |

*N.D.: not detected
